# The risk profile of childhood leukaemia in Greece: a nationwide case-control study.

**DOI:** 10.1038/bjc.1997.541

**Published:** 1997

**Authors:** E. Petridou, D. Trichopoulos, V. Kalapothaki, A. Pourtsidis, M. Kogevinas, M. Kalmanti, D. Koliouskas, H. Kosmidis, J. P. Panagiotou, F. Piperopoulou, F. Tzortzatou

**Affiliations:** Department of Hygiene and Epidemiology, Athens University Medical School, Greece.

## Abstract

The risk profile of childhood leukaemia in Greece was studied through a case-control investigation that included all 153 incident cases of the disease, ascertained throughout the country during 1993 and 1994, and two hospital controls for every case matched for gender, age and place of residence. The data were analysed using conditional logistic regression and the associations are expressed in terms of adjusted odds ratios (OR) and their 95% confidence intervals. Cases were born to mothers of a higher standard education, the OR for an increment of four schooling years being 1.48 (1.17-1.87) and had higher birth weight, the OR for an increment of 500g being 1.36 (1.04-1.77). Pet ownership and birth after a pregnancy with anaemia were associated with increased risk, the ORs being 2.18 (1.14-4.16) and 2.60 (1.39-4.86) respectively. From the frequency analyses, indicative inverse associations were found with birth order, household crowding and previous hospitalization with allergic diseases, whereas indicative positive associations were found with diabetes mellitus during pregnancy and with neonatal jaundice. Substantial or significant elevations were not found with respect to maternal smoking and coffee drinking during pregnancy, diagnostic radiography and ultrasonographic examinations or blood transfusions. A significant inverse association with maternal consumption of alcohol could be due to multiple comparisons, but a detrimental effect can probably be excluded. A non-significant positive association with total shots of viral vaccinations and a weak non-significant inverse association with breast feeding were also found. We interpret the findings of this study as being compatible with acute childhood leukaemia being linked with delayed development of herd immunity to fairly common infectious agents, in conjunction with accelerated perinatal and early post-natal growth.


British Journal of Cancer (1997) 76(9), 1241–1247
© 1997 Cancer Research Campaign

### The risk profile of childhood leukaemia in Greece: a nationwide case–control study

**E Petridou[1,2], D Trichopoulos[1,2], V Kalapothaki[1], A Pourtsidis[3], M Kogevinas[4], M Kalmanti[5], D Koliouskas[6], H Kosmidis[3], JP Panagiotou[7], F Piperopoulou[8] and F Tzortzatou[9]**

[1]Department of Hygiene and Epidemiology, Athens University Medical School, 75 M. Asias Str., Athens 11527, Greece; [2]Department of Epidemiology, Harvard School of Public Health, 677 Huntington Ave., Boston, MA 02115, USA; [3]Department of Pediatric Hematology–Oncology, A Kyriakou Children's Hospital, Thivon & Levadias Str., Athens 11527, Greece; [4]Institute Municipal d'Investigacio Medica IMIM, Aiguader 80, E-08003 Barcelona, Spain; [5]Department of Pediatric Hematology–Oncology, University Hospital, Heraklion 71500, Greece; [6]Department of Pediatric Hematology–Oncology, Ippocrateion General Hospital, 49 Constantinoupoleos Str., Salonica 54636, Greece; [7]Department of Pediatric, Hematology–Oncology, Agia Sophia Children's Hospital, Athens 11527 Greece; [8]Second Department of Pediatrics, Aristoteleion University of Salonica, AHEPA General Hospital, 1 S Kyriakidi Str., 54636 Salonica, Greece; [9]Unit of Childhood Hematology–Oncology, First Department of Pediatrics, Athens University Medical School, Agia Sophia Children's Hospital, Athens 11527, Greece

**Summary** The risk profile of childhood leukaemia in Greece was studied through a case–control investigation that included all 153 incident cases of the disease, ascertained throughout the country during 1993 and 1994, and two hospital controls for every case matched for gender, age and place of residence. The data were analysed using conditional logistic regression and the associations are expressed in terms of adjusted odds ratios (OR) and their 95% confidence intervals. Cases were born to mothers of a higher standard education, the OR for an increment of four schooling years being 1.48 (1.17–1.87) and had higher birth weight, the OR for an increment of 500 g being 1.36 (1.04–1.77). Pet ownership and birth after a pregnancy with anaemia were associated with increased risk, the ORs being 2.18 (1.14–4.16) and 2.60 (1.39–4.86) respectively. From the frequency analyses, indicative inverse associations were found with birth order, household crowding and previous hospitalization with allergic diseases, whereas indicative positive associations were found with diabetes mellitus during pregnancy and with neonatal jaundice. Substantial or significant elevations were not found with respect to maternal smoking and coffee drinking during pregnancy, diagnostic radiography and ultrasonographic examinations or blood transfusions. A significant inverse association with maternal consumption of alcohol could be due to multiple comparisons, but a detrimental effect can probably be excluded. A non-significant positive association with total shots of viral vaccinations and a weak non-significant inverse association with breast feeding were also found. We interpret the findings of this study as being compatible with acute childhood leukaemia being linked with delayed development of herd immunity to fairly common infectious agents, in conjunction with accelerated perinatal and early post-natal growth.

**Keywords:** childhood leukaemia; birthweight; herd immunity; pet ownership

The aetiology of childhood leukaemia remains an enigma. Ionizing radiation is an established cause (US National Academy of Sciences Committee on the Biological Effects of Ionizing Radiation, 1990), but it is unlikely to explain more than a small fraction of all cases (probably less than 20%). Epidemiological studies throughout the world have explored several groups of factors, and the collective evidence has recently been summarized (Chow et al, 1996; Linet & Cartwright, 1996). Infectious agents remain prime candidates because of the animal evidence and the role of HTLV-I in a rare form of leukaemia (Mueller, 1991), but opinions diverge as to whether a specific virus is implicated or whether common infectious agents are responsible, their effect conditioned by particular genotypes or certain patterns of herd immunity (Kinlen et al, 1990, 1995; MacMahon, 1992; Greaves and Alexander, 1993; Petridou et al, 1993; Kinlen, 1995; Greaves, 1997). Paradigms from other diseases have stimulated searches for prenatal viral (Curnen et al, 1974; Randolph and Heath, 1974; Fine et al, 1985) and chemical exposures (Cutler et al, 1986; Lowengart et al, 1987; Shu et al, 1988; Robison et al, 1989; Savitz and Chen, 1990; Zack et al, 1991), but these results have been collectively unconvincing (Chow et al, 1996). Research focusing on post-natal chemical exposures, including diet, vitamins and drugs (Shu et al, 1988; Olsen et al, 1994; Peters et al, 1994; Sarasua and Savitz, 1994) – with the possible exceptions of chemotherapy (Pui et al, 1991) and administration of growth hormone (Ritzen, 1993) – has also been inconclusive. Studies of correlates of perinatal growth, notably birth weight, have generated intriguing results, but the nature of the underlying processes and the preventive implications of these findings remain elusive (MacMahon and Newill, 1962; Daling et al, 1984; Robison et al, 1987; Zack et al, 1991). Types of radiation other than ionizing have also been investigated, including extremely low frequency electric and magnetic fields (ELF, EMF) (Oak Ridge Associated Universities, 1992; National Research Council, 1996), radiofrequency waves (Maskarinec, 1994), phototherapy (Ben-Sasson and Davis, 1992; Miller, 1992) and diagnostic ultrasounds (Shu et al, 1994), but the evidence is contradictory.

Certain correlation studies in Greece have provided evidence for an infectious aetiology of childhood leukaemia (Kinlen and Petridou, 1995; Petridou et al, 1996*a*) and an earlier case–control investigation that has no overlap with the present study also

*Received 20 February 1997*
*Revised 7 May 1997*
*Accepted 14 May 1997*

*Correspondence to:* E Petridou, Department of Hygiene and Epidemiology, Athens University Medical School, 75 M. Asias Str., Athens 11527, Greece pointed in the same direction (Petridou et al, 1993). During 1993–94 we undertook a nationwide study to ascertain the risk factors for childhood leukaemia in this country. A study protocol similar to that used in ongoing case–control investigations in other European countries was used. Blood samples were also collected, and sera and bone marrow slides have been preserved for the evaluation of any new hypothesis that may be proposed in the near future.

### SUBJECTS AND METHODS

During the 2-year period 1993–94, 153 bone marrow confirmed cases of childhood (aged 0–14 years) leukaemia were diagnosed through a nationwide network of childhood haematologists/oncologists giving an incidence of 41.8 cases per $10^{-6}$ person–years. This rate is similar to or slightly higher than rates observed in other developed countries. Of these cases, 136 (89%) were of the acute lymphoblastic type. Ascertainment of incident childhood leukaemia cases is thought to be virtually complete, with the exception of a few cases that were diagnosed and treated abroad (Petridou et al, 1996*a* and *b*). Nine additional children with leukaemia were not eligible for the study because they were not of Greek nationality.

For every case the first two hospital controls that met with preset matching criteria were enrolled in the study. Controls had to be hospitalized at the same time as the corresponding cases for acute conditions; be of the same gender and age (± 6 months for cases younger than 3 years and ± 12 months for older children) and, for urban areas, reside at the time of diagnosis in the same town or, for rural residents, at any village in the same geographic region.

The guardians of all cases (95% mothers) and controls (96% mothers) were informed of the study objectives and were asked to respond to an interviewer-administered questionnaire concerning sociodemographic, environmental, lifestyle and biomedical variables and to allow a blood sample to be taken from the child for serum preservation. There were no refusals from the guardians of the cases but the guardians of 14, otherwise eligible, controls (4%) were unable or unwilling to participate, and eight of them were replaced.

Measures of electric and magnetic field exposure were ascertained blindly for cases and controls through collaboration with the Public Power Corporation (PPC). Relevant results as well as those derived from serological tests will be reported separately. Frequency distributions of the different variables were examined initially and the data were then modelled using conditional logistic regression (Breslow and Day, 1980). Unconditional regression with additional terms for the matching factors generated very similar results. Given the weak overall associations, we opted against using stepwise regression because this could have left substantial residual confounding (MacMahon and Trichopolos, 1996).

### RESULTS

Table 1 presents frequency distributions of cases and controls by sociodemographic, lifestyle, environmental and biomedical variables. In the same table the bivariate associations are evaluated and the respective *P*-values, derived from $\chi^2$ with one degree of freedom, are given. A plus or minus sign indicates a positive or inverse association, respectively, considering in all instances the first of the indicated categories as unexposed or less exposed. The patterns in this table are indicative but they are not aetiologically interpretable in strict terms because of mutual confounding and the underlying matching.

Table 2 shows conditional logistic regression-derived, mutually adjusted odds ratios for childhood leukaemia in specific exposure categories or by specified increments in the study variables. There was a highly significant positive association with level of maternal education ($P$ = 0.001). It appears also that the risk of childhood leukaemia increases with maternal age at birth and sibship size and decreases with increasing birth order; however, these patterns are not statistically significant. Day care attendance and household crowding, proxies for infectious agent transmission at an early age, are associated with slightly reduced risk but again neither of these associations is statistically significant. Among lifestyle variables, pet ownership at any time before diagnosis (cases) or interview (controls) was associated with significantly increased risk ($P$ = 0.02), whereas consumption of small or moderate quantities of alcohol during pregnancy was associated with significantly reduced risk ($P$ = 0.03); it should be noted that no woman consumed during her pregnancy of the index child more than a glass of alcoholic beverages per day. Maternal tobacco smoking and coffee drinking were unrelated to childhood leukaemia risk, even though these exposures were evaluated during the restricted period of the index pregnancy.

None of the environmental exposures in Table 2 was significantly associated with childhood leukaemia risk; in fact diagnostic radiography, ultasonographs and ground floor residence (a proxy for radon exposure) were, if anything, inversely related to this risk.

Among the biomedical variables birthweight was, as expected, positively associated with childhood leukaemia ($P$ = 0.02) as was a history of anaemia during pregnancy ($P$ = 0.003). None of the other biomedical variables showed a significant association with disease risk, including a history of blood transfusion(s). It is of some interest that hospitalization for allergic diseases was inversely, albeit not significantly, associated with disease risk.

Introduction into the model presented in Table 2 of measures of exposure to magnetic fields from electrical wiring did not substantially affect the results in this table (data not shown).

### DISCUSSION

This study, despite including as many cases as have occurred in the country over the study period, is only of moderate size and the use of hospital controls is always a contentious issue. However, the use of hospital controls assured a high level of cooperation from the patients and their guardians, including permission for collection of blood samples. Population-based controls are in principle superior because they represent the study base, but refusal rates can be so high as to compromise the desired comparability between the case and control series (MacMahon and Trichopoulos, 1996). The ability of the present study to show the few established risk factors for childhood leukaemia argues in favour of the validity of other results, and control for socioeconomic status, as reflected in maternal education, should substantially reduce any social class-related selection bias in hospital admission procedures.

Most straightforward were the results of this study in relation to the environmental exposures considered, in that they were convincingly null. Neither the infrequent diagnostic radiography

 © Cancer Research Campaign 1997

**Table 1** Distribution of 153 childhood leukaemia cases and 300 age-, gender- and residence-matched controls by study variables

| Variable | Cases n | Cases % | Controls n | Controls % | P-value for trend (direction) |
|---|---|---|---|---|---|
| *Socioeconomic variables* | | | | | |
| Gender | | | | | Matched variable |
| Male | 85 | 55.6 | 168 | 56.0 | |
| Female | 68 | 44.4 | 132 | 44.0 | |
| Age (years) | | | | | Matched variable |
| ≥ 4 | 71 | 46.4 | 124 | 41.4 | |
| 5–9 | 50 | 32.7 | 108 | 36.0 | |
| ≥ 10 | 32 | 20.9 | 68 | 22.6 | |
| Residence | | | | | Matched variable |
| Urban | 82 | 53.6 | 173 | 57.7 | |
| Rural | 71 | 46.4 | 127 | 42.3 | |
| Maternal age (years) | | | | | 0.10 (+) |
| < 20 | 11 | 7.2 | 21 | 7.0 | |
| 20–24 | 42 | 27.5 | 102 | 34.0 | |
| 25–29 | 45 | 29.4 | 95 | 31.7 | |
| 30–34 | 40 | 26.1 | 61 | 20.3 | |
| ≤ 35 | 15 | 9.8 | 21 | 7.0 | |
| Maternal education (years) | | | | | < 0.001(+) |
| 6–9 | 51 | 33.3 | 157 | 52.3 | |
| 10–12 | 58 | 37.9 | 91 | 30.3 | |
| > 12 | 44 | 28.8 | 52 | 17.4 | |
| Sibship size | | | | | 0.12 (–) |
| 1 | 29 | 18.9 | 56 | 18.7 | |
| 2 | 99 | 64.7 | 166 | 55.3 | |
| ≥ 3 | 25 | 16.4 | 78 | 26.0 | |
| Birth order | | | | | 0.11 (–) |
| 1 | 71 | 46.4 | 129 | 43.0 | |
| 2 | 70 | 45.8 | 125 | 41.7 | |
| ≥ 3 | 12 | 7.8 | 46 | 15.3 | |
| Persons per room | | | | | 0.05 (–) |
| < 1.25 | 15 | 9.8 | 16 | 5.3 | |
| 1.25–1.54 | 39 | 25.5 | 68 | 22.7 | |
| 1.55–1.99 | 62 | 40.5 | 126 | 42.0 | |
| ≥ 2 | 37 | 24.2 | 90 | 30.0 | |
| Day care (ever) | | | | | 0.81 (+) |
| No | 91 | 59.5 | 182 | 60.7 | |
| Yes | 62 | 40.5 | 118 | 39.3 | |
| *Lifestyle variables* | | | | | |
| Maternal smoking | | | | | 0.74 (+) |
| No | 76 | 49.7 | 154 | 51.3 | |
| Yes | 77 | 50.3 | 146 | 48.7 | |
| Maternal alcohol consumption[a] | | | | | 0.11 (–) |
| No | 67 | 43.8 | 108 | 36.0 | |
| Yes | 86 | 56.2 | 192 | 64.0 | |
| Maternal coffee drinking[b] | | | | | 0.27 (–) |
| No | 60 | 39.2 | 102 | 34.0 | |
| Yes | 93 | 60.8 | 198 | 66.0 | |
| Breast feeding | | | | | 0.78 (+) |
| No | 52 | 34.0 | 106 | 35.3 | |
| Yes | 101 | 66.0 | 194 | 64.7 | |
| Pet ownership[c] | | | | | 0.06 (+) |
| No | 120 | 78.4 | 256 | 85.3 | |
| Yes | 33 | 21.6 | 44 | 14.7 | |
| *Environmental variables* | | | | | |
| Diagnostic irradiation during pregnancy | | | | | 0.83 (–) |
| No | 148 | 96.7 | 289 | 96.3 | |
| Yes | 5 | 3.3 | 11 | 3.7 | |
| Diagnostic ultrasound during pregnancy | | | | | 0.94 (+) |
| No | 53 | 34.6 | 105 | 35.0 | |
| Yes | 100 | 65.4 | 195 | 65.0 | |
| Residential floor | | | | | 0.21 (–) |
| ≥ two floors | 103 | 67.3 | 184 | 61.3 | |
| < two floors | 50 | 32.7 | 116 | 38.7 | |

© Cancer Research Campaign 1997

**Table 1** Cont.

| Variable | Cases | | Controls | | P-value for trend (direction) |
|---|---|---|---|---|---|
| | *n* | % | *n* | % | |
| House heating | | | | | 0.41 (+) |
| Non-electrical | 113 | 73.9 | 232 | 77.3 | |
| Electrical | 40 | 26.1 | 68 | 22.7 | |
| Hair dryer use | | | | | 0.41 (+) |
| No | 94 | 61.4 | 196 | 65.3 | |
| Yes | 59 | 38.6 | 104 | 34.7 | |
| *Biomedical variables* | | | | | |
| Anaemia during pregnancy | | | | | 0.01 (+) |
| No | 81 | 52.9 | 195 | 65.0 | |
| Yes | 72 | 47.1 | 105 | 35.0 | |
| Diabetes melitus during pregnancy | | | | | 0.21 (+) |
| No | 150 | 98.0 | 298 | 99.3 | |
| Yes | 3 | 2.0 | 2 | 0.7 | |
| Birth weight (g) | | | | | 0.18 (+) |
| <2500 | 4 | 2.6 | 19 | 6.3 | |
| 2500–2999 | 28 | 18.3 | 51 | 17.0 | |
| 3000–3499 | 57 | 37.3 | 113 | 37.7 | |
| 3500–3999 | 45 | 29.4 | 93 | 31.0 | |
| ≥ 4000 | 19 | 12.4 | 24 | 8.0 | |
| Neonatal jaundice | | | | | 0.80 (+) |
| No | 125 | 81.7 | 248 | 82.7 | |
| Yes | 28 | 18.3 | 52 | 17.3 | |
| Blood transfusion(s)[c] | | | | | 0.83 (–) |
| No | 148 | 96.7 | 289 | 96.3 | |
| Yes | 5 | 3.3 | 11 | 3.7 | |
| Allergic disease (hospitalized) | | | | | 0.04 (–) |
| No | 150 | 98.0 | 281 | 93.7 | |
| Yes | 3 | 2.0 | 19 | 6.3 | |
| Total DTP shots[d] | | | | | 0.36 (+) |
| 0 | 14 | 9.1 | 49 | 16.3 | |
| 1–8 | 27 | 17.7 | 42 | 14.0 | |
| 9–11 | 37 | 24.2 | 51 | 17.0 | |
| 12–13 | 36 | 23.5 | 97 | 32.4 | |
| ≥14 | 39 | 25.5 | 61 | 20.3 | |
| BCG vaccination | | | | | 0.76 (+) |
| No | 131 | 85.6 | 260 | 86.7 | |
| Yes | 22 | 14.4 | 40 | 13.3 | |
| Total viral vaccination shots[e] | | | | 0.09 (+) | |
| 0 | 15 | 9.8 | 51 | 17.0 | |
| 1–8 | 14 | 9.2 | 30 | 10.0 | |
| 9–12 | 35 | 22.9 | 65 | 21.7 | |
| 13–15 | 51 | 33.3 | 81 | 27.0 | |
| ≥ 16 | 38 | 24.8 | 73 | 24.3 | |

[a]Two or more glasses per week. [b]Three or more cups per week. [c]Ever, before diagnosis. [d]Diphtheria, pertussis, tetanus vaccines; each antigen is counted as a distinct shot. [e]Measles, mumps, rubella, hepatitis B vaccines; each antigen is counted as a distinct shot.

during pregnancy nor the common ultrasound monitoring of pregnancy were related to childhood leukaemia, nor was there evidence that residence at ground level, a proxy for radon exposure, was a risk factor in this data set. Diagnostic irradiation, as currently practised in late pregnancy using modern technology has minimal effect on the risk of childhood leukaemia (Mole, 1990) and the proxy variable for radon exposure used in this study is too crude to capture all but sizable risk elevations (Stjernfeld et al, 1987). Moreover, most studies that have investigated ultrasonography in relation to childhood leukaemia have reported no association. Electrical heating and the use of hair dryers both generate high intensity ELF-magnetic fields, but the associations noted were minimal and far from significant, in accordance with the majority of the evidence on the relationship of such fields to childhood leukaemia (National Radiological Protection Board, 1994; National Research Council, 1996).

Two studies have reported that prenatal exposure to tobacco compounds may increase the risk of childhood leukaemia (John et al, 1991; Cocco et al, 1996), but the results of our study provide little support for this suggestion. Some studies have reported an increased risk for certain subgroups of childhood leukaemia in relation to maternal consumption of alcohol (Severson et al, 1993; Van Duijn et al, 1994), but again our findings are not compatible with this suggestion, again in accordance with the majority of epidemiological evidence (Chow et al, 1996). We do not interpret the statistically significant inverse association of maternal alcohol consumption on childhood leukaemia risk in causal terms; it is far more likely to be a chance phenomenon generated by the multiple comparisons undertaken in the analysis. In agreement with most previous investigations, breast feeding was not significantly associated with disease risk (Hartley et al, 1988, Van Duijn et al, 1988). We also found no evidence that coffee drinking, in the modest

 *© Cancer Research Campaign 1997*

**Table 2** Multiple logistic regression-derived, mutually adjusted odds ratios (ORs) and 95% confidence intervals (95% CI) for childhood leukaemia in relation to specified exposures

| Variable | Category or increment | OR | 95% CI | *P*-value |
|---|---|---|---|---|
| *Sociodemographic variables* | | | | |
| Maternal age at birth | 5 years | 1.19 | 0.93–1.52 | 0.17 |
| Maternal education | ~4 years | 1.48 | 1.17–1.87 | 0.001 |
| Sibship size | one | 1.40 | 0.88–2.23 | 0.16 |
| Birth order | one | 0.74 | 0.48–1.15 | 0.18 |
| Persons per room | ~0.4 | 0.83 | 0.63–1.11 | 0.21 |
| Day care | ever (vs never) | 0.83 | 0.51–1.37 | 0.46 |
| *Lifestyle variables* | | | | |
| Maternal smoking | yes (vs no) | 1.19 | 0.73–1.93 | 0.49 |
| Maternal alcohol consumption[a] | yes (vs no) | 0.57 | 0.34–0.95 | 0.03 |
| Maternal coffee drinking[b] | yes (vs no) | 0.89 | 0.55–1.46 | 0.65 |
| Breast feeding | yes (vs no) | 0.85 | 0.52–1.41 | 0.54 |
| Pet ownership[c] | yes (vs no) | 2.18 | 1.14–4.16 | 0.02 |
| *Environmental variables* | | | | |
| Pregnancy radiography | yes (vs no) | 0.84 | 0.25–2.78 | 0.77 |
| Pregnancy ultrasound | yes (vs no) | 0.74 | 0.41–1.32 | 0.30 |
| Residential floor | ground level (vs other) | 0.85 | 0.50–1.43 | 0.53 |
| House heating | electrical (vs non-electrical) | 1.04 | 0.60–1.79 | 0.89 |
| Hair dryer use | yes (vs no) | 1.37 | 0.79–2.37 | 0.27 |
| *Biomedical variables* | | | | |
| Anaemia during pregnancy | yes (vs no) | 2.60 | 1.39–4.86 | 0.003 |
| Diabetes melitus during pregnancy | yes (vs no) | 2.99 | 0.30–29.56 | 0.35 |
| Birthweight | 500 g | 1.36 | 1.04–1.77 | 0.02 |
| Neonatal jaundice | yes (vs no) | 1.44 | 0.74–2.78 | 0.28 |
| Blood transfusions(s)[c] | yes (vs no) | 0.22 | 0.38–4.93 | 0.64 |
| Allergic disease (hospitalized) | yes (vs no) | 0.36 | 0.09–1.43 | 0.15 |
| Total DTP shots[d] | ~3 | 0.97 | 0.71–1.32 | 0.83 |
| BCG vaccination | yes (vs no) | 1.44 | 0.66–3.13 | 0.36 |
| Total viral vaccination shots[e] | ~3 | 1.23 | 0.91–1.66 | 0.16 |

[a]Two or more glasses per week. [b]Three or more cups per week. [c]Ever, before diagnosis. [d]Diphtheria, pertusis, tetanus vaccines: each antigen is counted as a distinct shot. [e]Measles, mumps, rubella, hepatitis B vaccines: each antigen is counted as a distinct shot.

quantities usually consumed during pregnancy in Greece, affects childhood leukaemia risk.

Pet ownership is not considered among the lay public in Greece as a possible risk factor for childhood leukaemia nor was the hypothesis considered as credible by the investigators during the planning and implementation of the study; it is therefore unlikely that information bias is responsible for the statistically significant positive association, although chance is again a possible explanation. It is worth noting, however, that ownership of a cat, a common domestic animal in Greece, has also been implicated in the aetiology of childhood leukaemia in earlier investigations (Bross and Gibson, 1970; Buckley et al, 1994).

Among the sociodemographic variables investigated none emerged as a powerful or statistically significant risk predictor, with the exception of a higher standard of maternal education, the most reliable indicator of socioeconomic status in Greece (Trichopoulos, 1982). Many studies have supported a link of childhood leukaemia with higher socioeconomic status in several populations through use of different social class indicators (Greenberg and Shuster, 1985; Van Steensel-Moll et al, 1986; Kaye et al, 1991). The positive association of maternal age at birth with disease risk, although not statistically significant, is compatible with the results of most earlier investigations that addressed this issue, (MacMahon and Newill, 1962; Buckley et al, 1994; Chow et al, 1996); null results, however, have also been reported from several major investigations (Robison et al, 1987; Zack et al, 1991).

The inverse associations of birth order, household crowding and day care attendance with childhood leukaemia risk are statistically non-significant but they all point to a higher risk of the disease with delayed exposure to infectious agents. These findings are consistent with the infection hypotheses of childhood leukaemia proposed by Kinlen et al (1990), Greaves and Alexander (1993) and other investigators (Van Steensel et al, 1986; MacMahon, 1992; Petridou et al, 1993; Kinlen, 1995; Kinlen et al, 1995). Our limited data on blood transfusion do not support a blood-transmitted agent being responsible for childhood leukaemia, in keeping with the larger study of Memon and Doll (1994). We have found no evidence that BCG and DTP vaccinations or immunization against common viral childhood infections conveyed protection, in spite of some previous reports (Haro, 1986; Kneale et al, 1986; Hartley et al, 1988; Nishi and Miyake, 1989). In fact, our data, if anything, indicate the opposite. The inverse association with allergic diseases is statistically non-significant and could be due to chance, but it deserves some attention in view of the fact that inverse associations have also been reported for other forms of cancer (Jain et al, 1991; Bueno et al, 1992).

The positive association between birth weight and the risk for childhood leukaemia has been reported by many investigators

© Cancer Research Campaign 1997

(MacMahon and Newill, 1962; Fasal et al, 1971; Hirayama, 1980; Daling, 1984; Robison et al, 1987) and, notwithstanding a few null reports, the association appears to be genuine. The credibility of this association is supported by the plausible, albeit weak, leukaemogenic effect of growth hormone and the reported link between height and acute lymphoblastic leukaemia (Broomhall et al, 1983). Additional evidence in support of an association between some growth processes and risk of childhood leukaemia can be found in two other results of the present investigation: the positive association with anaemia during pregnancy, because anaemia is associated with increased placental weight (Godfrey et al, 1991), and the non-significant, positive association with diabetes mellitus during pregnancy, because of the intimate link of diabetes mellitus with IGF1 and growth in general. The non-significant association with neonatal jaundice in our data could be due to chance, but a similar association has been reported from an American investigation (Buckley et al, 1994) and a Swedish study (Zack et al, 1991). The pathophysiology of neonatal jaundice is not fully understood but a link with oestrogens that have growth-promoting potential has been suggested (Lauritzen et al, 1966; Robine et al, 1988).

It is not easy to integrate the results of this investigation into a coherent pattern when taking into account the collective evidence from earlier investigations. Nevertheless, our data appear to be compatible with acute childhood leukaemia being linked with a delayed development of herd immunity to fairly common infectious agents, in conjunction with accelerated perinatal and early post-natal growth. This hypothesis could explain the positive associations with maternal education, birth weight and anaemia during pregnancy, as well as the inverse associations with birth order, household crowding and day care attendance. The multitude and unpredictability of factors that shape herd immunity and determine growth could explain why the odds ratio for each of these factors deviates only modestly from the null value. Pet ownership may be important only in particular settings, depending on epizootic parameters, but the issue deserves more attention. The modest size of the present study did not allow investigation of acute childhood leukaemia by histological subtype, but the risk factor profile that is indicated is clearly heavily weighted by the dominant type of acute lymphoblastic leukaemia.

### ACKNOWLEDGEMENTS

This study was supported in part by the Europe Against Cancer Program (DGV) of the European Union. We are indebted to all treating physicians, the children and their guardians for their collaboration in this study.

### REFERENCES

Ben-Sasson SA and Davis DL (1992) Neonatal exposure to protoporphyrin-activating lighting as a contributing cause of childhood acute lymphoblastic leukemia. *Cancer Causes Control* **3**: 383–387

Breslow NE and Day NE (1980) *Statistical Methods in Cancer Research* Vol. I. *The Analysis of Case–Control Studies*. IARC Scientific Publications No 32. IARC: Lyon

Broomhall J, May R, Lilleyman JS and Milner RDG (1983) Height and lymphoblastic leukemia. *Arch Dis Child* **58**: 300–301

Bross ID and Gibson R (1970) Cats and childhood leukemia. *J Med* **1**: 180–187

Buckley JD, Buckley CM, Ruccione K, Sather HN, Waskerwitz MJ, Woods WG and Robison LL (1994) Epidemiological characteristics of childhood acute lymphocytic leukemia. Analysis by immunophenotype. The children's cancer group. *Leukemia* **8**: 856–864

Bueno De Mesquita HB, Maisonneuve P, Moerman CJ and Walker AM (1992) Aspects of medical history and exocrine carcinoma of the pancreas. A population-based case–control study in the Netherlands. *Int J Cancer* **52**: 17–23

Chow W-H, Linet MS, Liff JM and Greenberg RS (1996) Cancers in children. In *Cancer Epidemiology and Prevention*, 2nd edn. Schottenfeld D and Fraumeni JF Jr. (eds), pp. 1331–1369. Oxford University Press: New York.

Cocco P, Rapallo M, Targhetta R, Biddau PF and Fadda D (1996) Analysis of risk factors in a cluster of childhood acute lymphoblastic leukemia. *Arch Environ. Health* **51**: 242–244

Curnen MG, Varma AA, Christine BW and Turgeon LR (1974) Childhood leukemia and maternal infectious diseases during pregnancy. *J Natl Cancer Inst* **53**: 943–947

Cutler JJ, Parker GS, Rosen S, Prenney B, Healey R and Caldwell GG (1986) Childhood leukemia in Woburn, Massachusetts. *Public Health Rep* **101**: 201–205

Daling JR, Starzyk P, Olshan AF and Weiss NS (1984) Birth weight and the incidence of childhood cancer. *J Natl Cancer Inst* **72**: 1039–1041

Fasal E, Jackson EW and Klauber MR (1971) Birth characteristics and leukemia in childhood. *J Natl Cancer Inst* **47**: 501–509

Fine EM, Adelstein AM, Snowman J, Clarkson JA and Evans SM (1985) Longterm effects of exposure to viral infections in utero. *Br Med J* **290**: 509–511

Godfrey KM, Redman CWG, Barker DJP and Osmond C (1991) The effect of maternal anaemia and iron deficiency on the ratio of fetal weight to placental weight. *Br J Obstet Gynecol* **98**: 886–891

Greaves MF (1997) Aetiology of acute leukaemia. *Lancet* **349**: 344–349

Greaves MF and Alexander FE (1993) An infectious etiology for common acute lymphoblastic leukemia in childhood? *Leukemia* **7**: 349–360

Greenberg RS and Shuster JL Jr (1985) Epidemiology of cancer in children. *Epidemiol Rev* **7**: 22–48

Haro AS (1986) The effect of BCG-vaccination and tuberculosis on the risk of leukemia. *Dev Biol Stand* (Part A) **58**: 433–449

Hartley AL, Birch JM, McKinney PA, Blair V, Teare MD, Carrette J, Mann JR, Stiller CA, Draper GJ, Johnston HC, Cartwright RA and Waterhouse JAH (1988) The inter-regional epidemiological study of childhood cancer (IRESCC): past medical history in children with cancer. *J Epidemiol Commun Health* **42**: 235–242

Hirayama T (1980) Descriptive and analytical epidemiology of childhood malignancy in Japan. In *Recent Advances in Management of Children with Cancer*, Kobayashi N. (ed.), pp. 27–43. The Children's Cancer Association of Japan: Tokyo

Jain M, Howe GR, St Louis P and Millar AB (1991) Coffee and alcohol as determinants of risk of pancreas cancer: a case–control study from Toronto. *Int J Cancer* **47**: 384–389

John EM, Savitz DA and Sandler DP (1991) Prenatal exposure to parents' smoking and childhood cancer. *Am J Epidemiol* **133**: 123–132

Kaye SA, Robison LL, Smithson WA, Gunderson P, King FL and Neglia JP (1991) Maternal reproductive history and birth characteristics in childhood acute lymphoblastic leukemia. *Cancer* **68**: 1351–1355

Kinlen LJ (1995) Epidemiological evidence for an infective basis in childhood leukaemia (Editorial). *Br J Cancer* **71**: 1–5

Kinlen LJ and Petridou E (1995) Childhood leukaemia and rural population movements: Greece, Italy, and other countries. *Cancer Causes Control* **6**: 445–451

Kinlen LJ, Clarke K and Hudson C (1990) Evidence from population mixing in British New Towns 1946–85 of an infective basis for childhood leukemia. *Lancet* **336**: 577–582

Kinlen LJ, Dickson M and Stiller CA (1995) Childhood leukaemia and non-Hodgkin's lymphoma near large rural construction sites, with a comparison with Sellafield nuclear site. *Br Med J* **310**: 763–768

Kneale GW, Stewart AM and Kinnier-Wilson LM (1986) Immunizations against infectious diseases and childhood cancers. *Cancer Immunol Immunother* **21**: 129–132

Lauritzen C and Lehmann WD (1966) The importance of steroid hormones in the pathogenesis of hyper-bilirubinemia and neonatal jaundice (German). *Z. Kinderheilkd* **95**:143–154

Linet MS and Cartwright RA (1996) The leukemias. In *Cancer Epidemiology and Prevention*, 2nd edn. Schottenfeld D and Fraumeni JF Jr. (eds), pp. 841–892. Oxford University Press: New York

Lowengart RA, Peters JM, Cicioni C, Buckley J, Bernstein L, Preston-Martin S and Rappaport E (1987) Childhood leukemia and parents' occupational and home exposures. *J Natl Cancer Inst* **79**: 39–46

MacMahon B (1992) Is acute lymphoblastic leukemia in children virus-related? *Am J Epidemiol* **136**: 916–924

 © Cancer Research Campaign 1997

MacMahon B and Newill VA (1962) Birth characteristics of children dying of malignant neoplasms. *J Natl Cancer Inst* **28**: 231–244
MacMahon B and Trichopoulos D (1996) *Epidemiology: Principles and Methods* (2nd edn). Little Brown & Co: Boston
Maskarinec G, Cooper J and Swygert L (1994) Investigation of increased incidence in childhood leukemia near radio towers in Hawaii: preliminary observations. *J Environ Pathol Toxicol Oncol* **13**: 33–37
Memon A and Doll R (1994) A search for unknown blood-borne oncogenic viruses. *Int J Cancer* **58**: 366–368
Miller RW (1992) Childhood leukemia and neonatal exposure to lighting in nurseries. *Cancer Causes Control* **3**: 581–582
Mole RH (1990) Childhood cancer after prenatal exposure to diagnostic x-ray examinations in Britain. *Br J Cancer* **62**: 152–168
Mueller N (1991) The epidemiology of HTLV-I infection. *Cancer Causes Control* **2**: 37–52
National Radiological Protection Board (1994) *Electromagnetic Fields and Risk of Cancer.* Vol. 5. pp. 77–81. National Radiological Protection Board: Chilton, Didcot, UK
National Research Council (1996) *Possible Health Effects of Exposure to Residential Electric and Magnetic Fields.* National Academy Press: Washington DC
Nishi M and Miyake H (1989) A case–control study on non-T-cell acute lymphoblastic leukemia of children in Hokkaido, Japan. *J Epidemiol Commun Health* **43**: 352–355
Oak Ridge Associated Universities (1992) *Health Effects of Low Frequency Electric and Magnetic Fields*. Publ. no. ORAU 92/F8. Committee in Interagency Radiation Research and Policy Coordination, Oak Ridge Associated Universities: Oak Ridge, TN, USA
Olsen JH, Hertz H, Blinkenberg K and Verder H (1994) Vitamin K regimens and incidence of childhood cancer in Denmark. *Br Med J* **308**: 895–896
Peters JM, Preston-Martin S, London SJ, Bowman JD, Buckley JD and Thomas DC (1994) Processed meats and risk of childhood leukemia (California, USA). *Cancer Causes Control* **5**: 195–202
Petridou E, Kassimos D, Kalmanti M, Kosmidou H, Haidas S, Flytzani V, Tong D and Trichopoulos D (1993) Age of exposure to infections and childhood leukemia risk. *Br Med J* **307**: 774
Petridou E, Revinthi K, Alexander FE, Haidas S, Koliouskas D, Kosmidis H, Piperopoulou F, Tzortzatou F and Trichopoulos D (1996*a*) Space–time clustering of childhood leukaemia in Greece: evidence supporting a viral aetiology. *Br J Cancer* **73**: 1278–1283
Petridou E, Trichopoulos D, Dessypris N, Flytzani V, Haidas S, Kalmanti M, Koliouskas D, Kosmidis H, Piperopoulou F and Tzortzatou F (1996*b*) Infant leukemia after in utero exposure to radiation from Chernobyl: evidence from Greece. *Nature* **382**: 352–353
Pui C-H, Ribeiro RC, Hancock ML, Rivera GK, Evans WE, Raimondi SC, Head DR, Behm FG, Mahmoud MH, Sandlund JT and Crist WM (1991) Acute myeloid leukemia in children treated with epipophyllotoxins for acute lymphoblastic leukemia. *N Engl J Med* **325**: 1682–1687
Randolph VL and Heath CW Jr (1974) Influenza during pregnancy in relation to subsequent childhood leukemia and lymphoma. *Am J Epidemiol* **100**: 399–409
Ritzen EM (1993) Does growth hormone increase the risk of malignancies? *Horm Res* **39**: 99–101
Robine N, Relier JP and Le Bars S (1988) Urocytogram, an index of maturity in premature infants. *Biol Neonate* **54**: 93–99
Robison LL, Codd M, Gunderson P, Neglia JP, Smithson WA and King FL (1987) Birth weight as a risk factor for childhood acute lymphoblastic leukemia. *Pediatr Hematol Oncol* **4**: 63–72
Robison LL, Buckley JD, Daigle AE, Wells R, Benjamin D, Arthur DC and Hammond GD (1989) Maternal drug use and risk of childhood nonlymphoblastic leukemia among off-spring. *Cancer* **63**: 1904–1911
Sarasua S and Savitz DA (1994) Cured and broiled meat consumption in relation to childhood cancer: Denver, Colorado (United States). *Cancer Causes Control* **5**: 141–148
Savitz DA and Chen J (1990) Parental occupation and childhood cancer: review of epidemiologic studies. *Environ Health Perspect* **88**: 325–337
Severson RK, Buckley JD, Woods WG, Benjamin D and Robison LL (1993) Cigarette smoking and alcohol consumption by parents of children with acute myeloid leukemia: an analysis within morphological subgroups – a report from the Childrens Cancer Group. *Cancer Epidemiol Biomark Prev* **2**: 433–439
Shu XO, Gao YT, Brinton LA, Linet MS, Tu JT, Zheng W and Fraumeni JF Jr (1988) A population-based case–control study of childhood leukemia in Shanghai. *Cancer* **62**: 635–644
Shu XO, Jin F, Linet MS, Zheng W, Clemens J, Mills J and Gao YT (1994) Diagnostic x-ray and ultra-sound exposure and risk of childhood cancer. *Br J Cancer* **70**: 531–536
Stjernfeld M, Samnelsson L and Ludvigsson J (1987) Radiation in dwellings and cancer in children. *Pediatr Hematol Oncol* **4**: 55–61
Trichopoulos D (1982) *Epidemiology, Principles and Methods* (in Greek). Parisianos (ed.), p. 555. Parisianos: Athens, Greece
US National Academy of Sciences Committee on the Biological Effects of Ionizing Radiation (1990) Health effects of exposure to low levels of ionizing radiation. 1990: BEIR-V Report. US NAS Washington
Van Duijn CA, Van Steensel-Moll HA, Van Der Does-VD-Berg A, Van Wering ER, Van Zanen GE, Valkenburg HA and Rammeloo JA (1988) Re: Infant feeding and childhood cancer. *Lancet* **2**: 796–797
Van Duijn CA, Van Steensel-Moll HA, Coebergh JW and Van Zanen GE (1994) Risk factors for childhood acute non-lymphocytic leukemia: an association with maternal alcohol consumption during pregnancy? *Cancer Epidemiol Biomarkers Prevention* **3**: 457–460
Van Steensel-Moll HA, Valkenburg HA and Van Zanen GE (1985) Childhood leukemia and parental occupation: a register-based case-control study. *Am J Epidemiol* **121**: 216–224
Van Steensel-Moll HA, Valkenburg HA and Van Zanen GE (1986) Childhood leukemia and infectious diseases in the first year of life: a register-based case–control study. *Am J Epidemiol* **124**: 590–594
Zack M, Adami H-O and Ericson A (1991) Maternal and perinatal risk factors for childhood leukemia. *Cancer Res* **51**: 3696–3701

© Cancer Research Campaign 1997


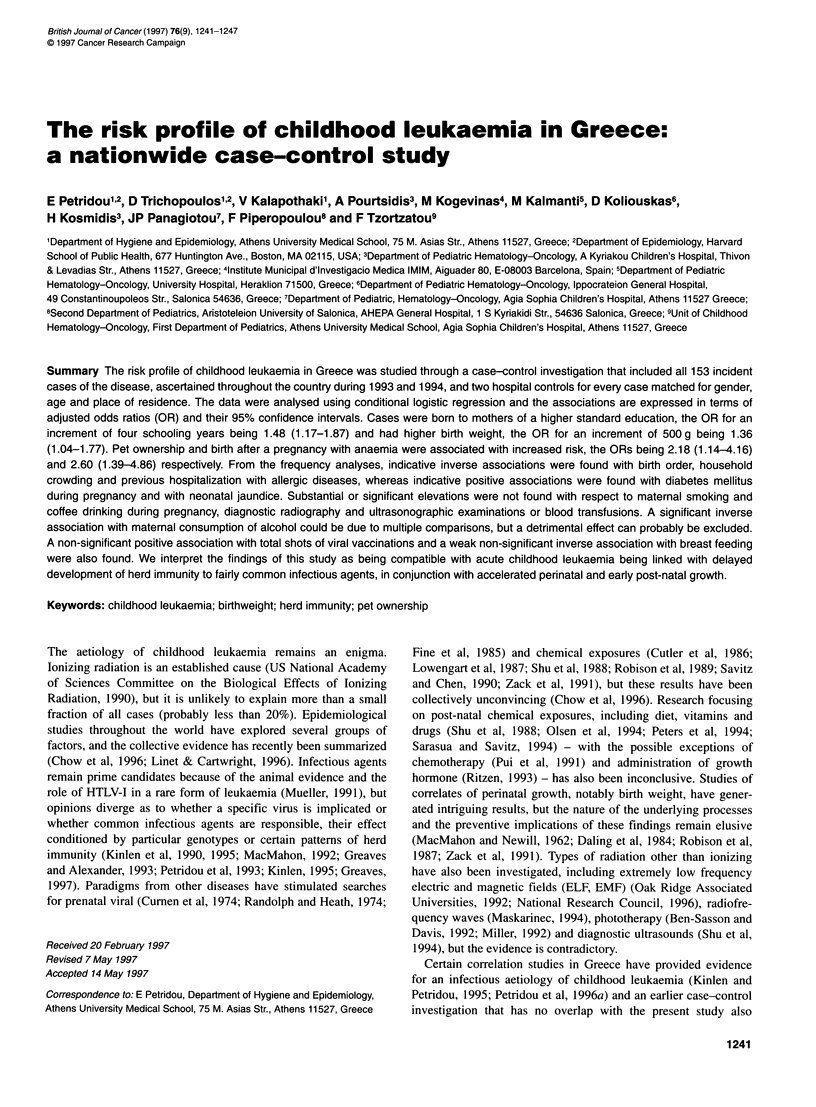

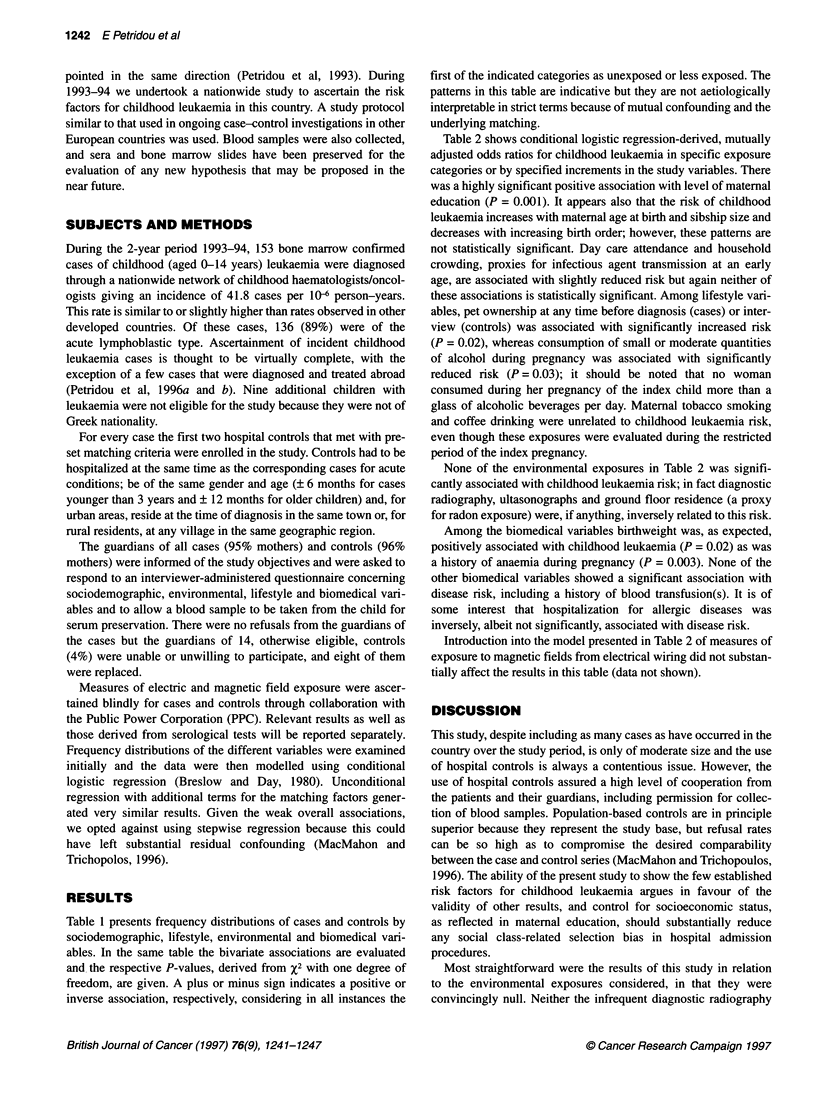

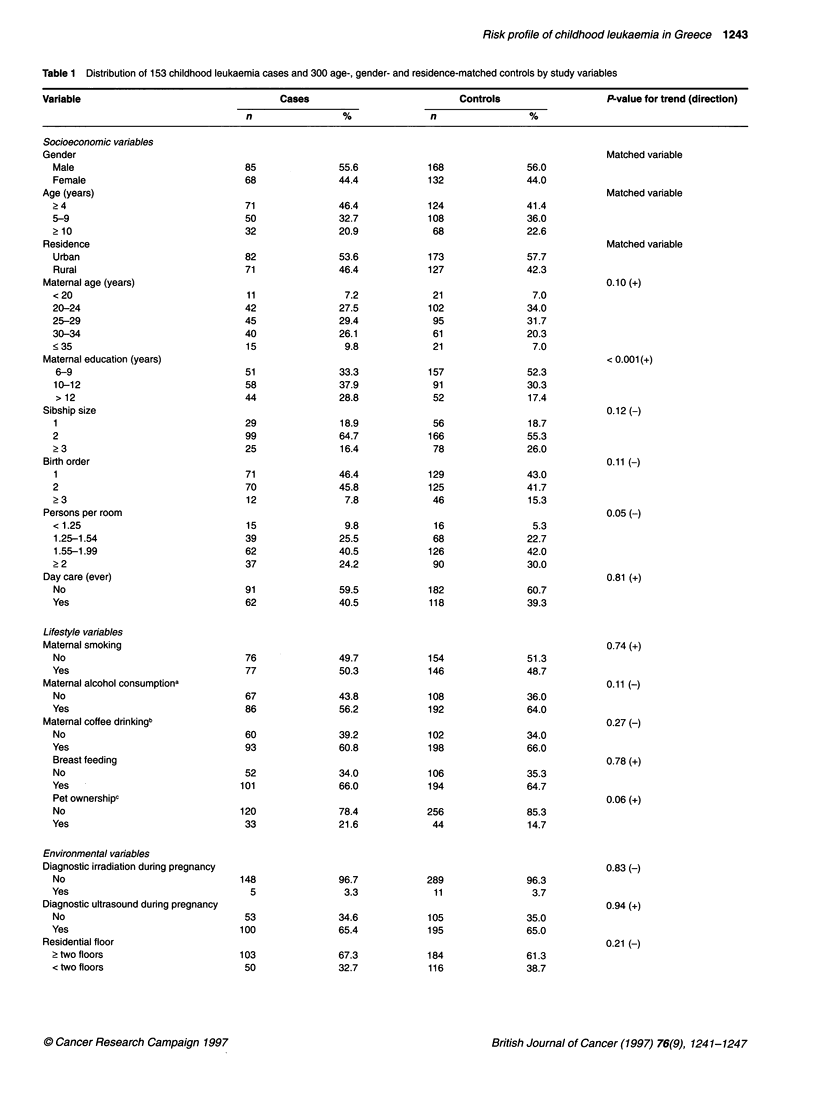

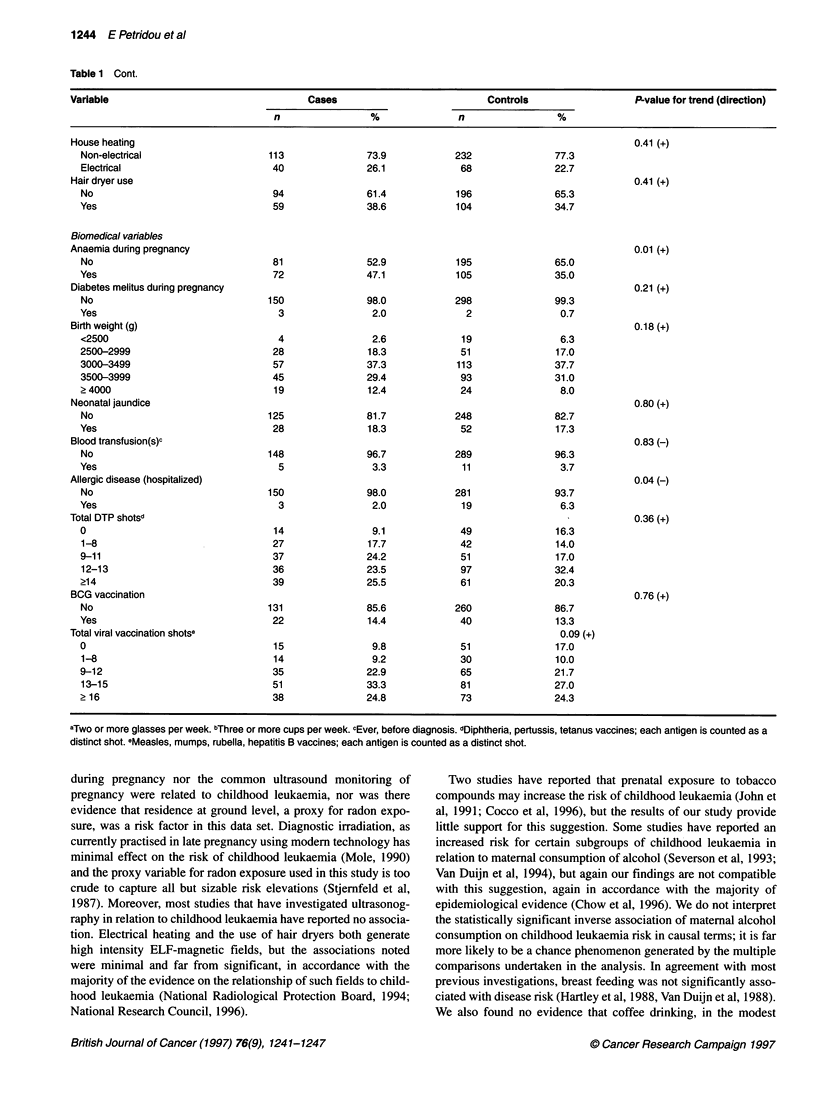

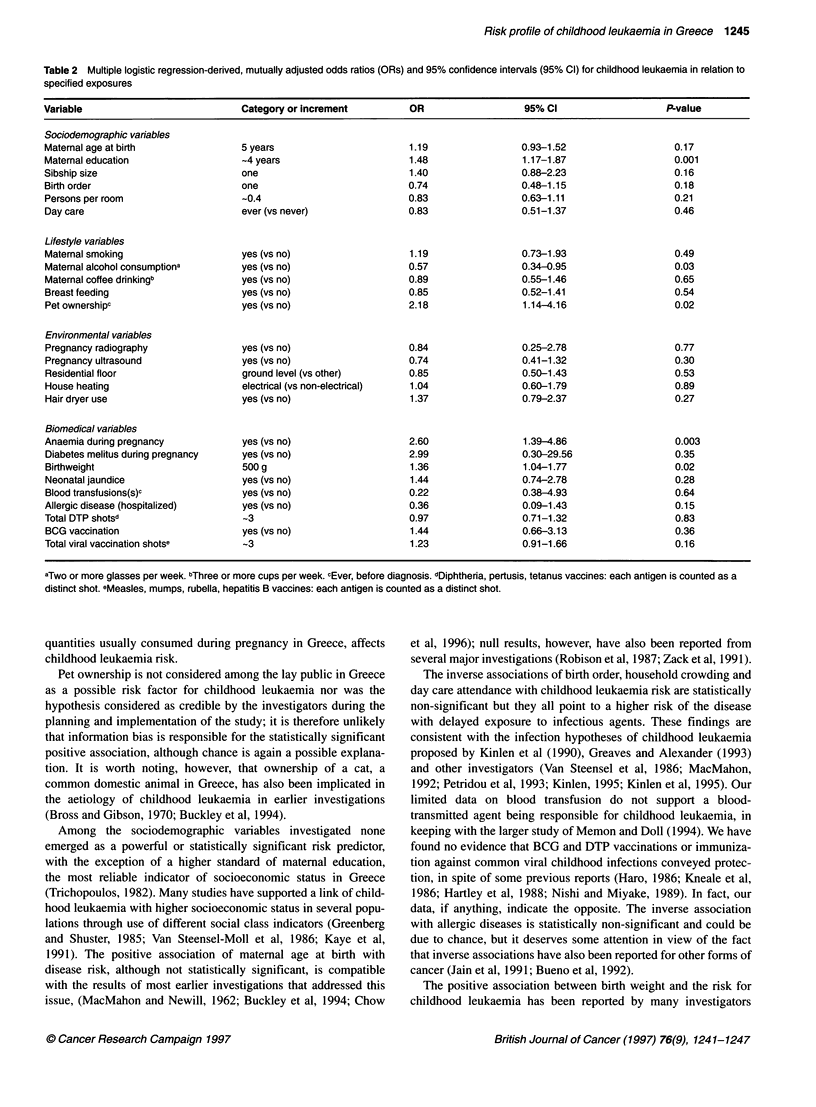

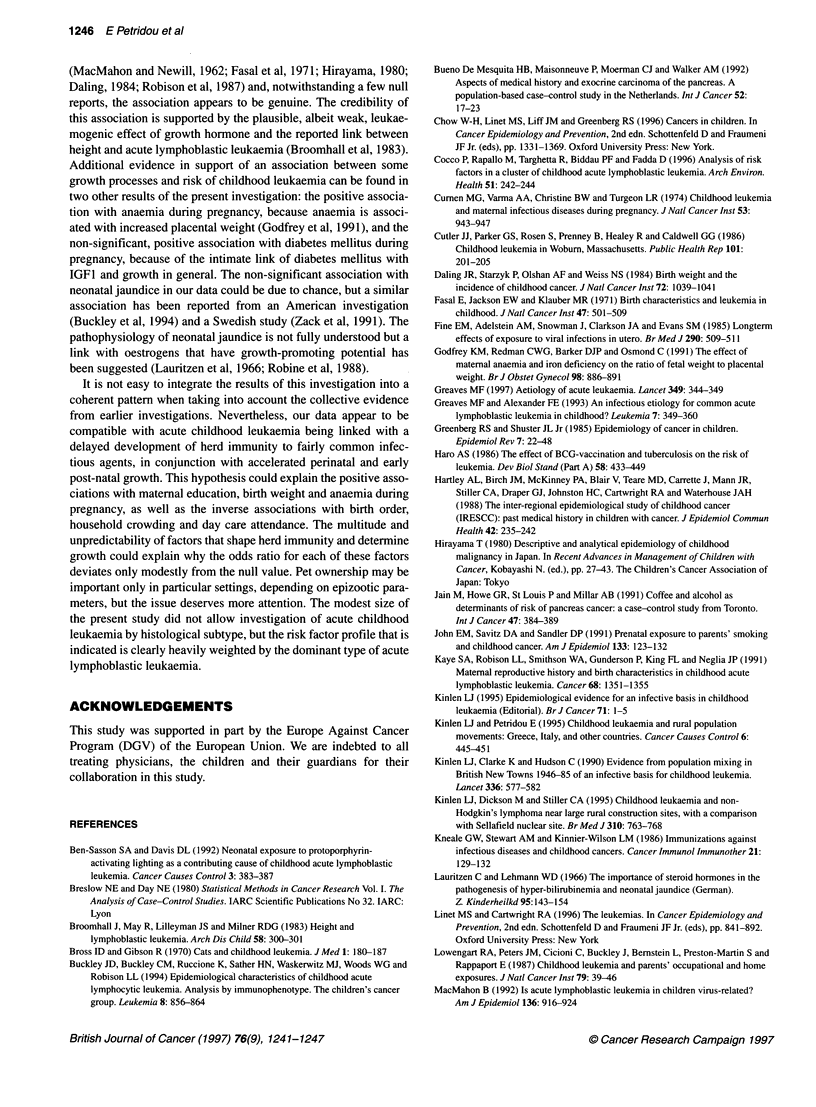

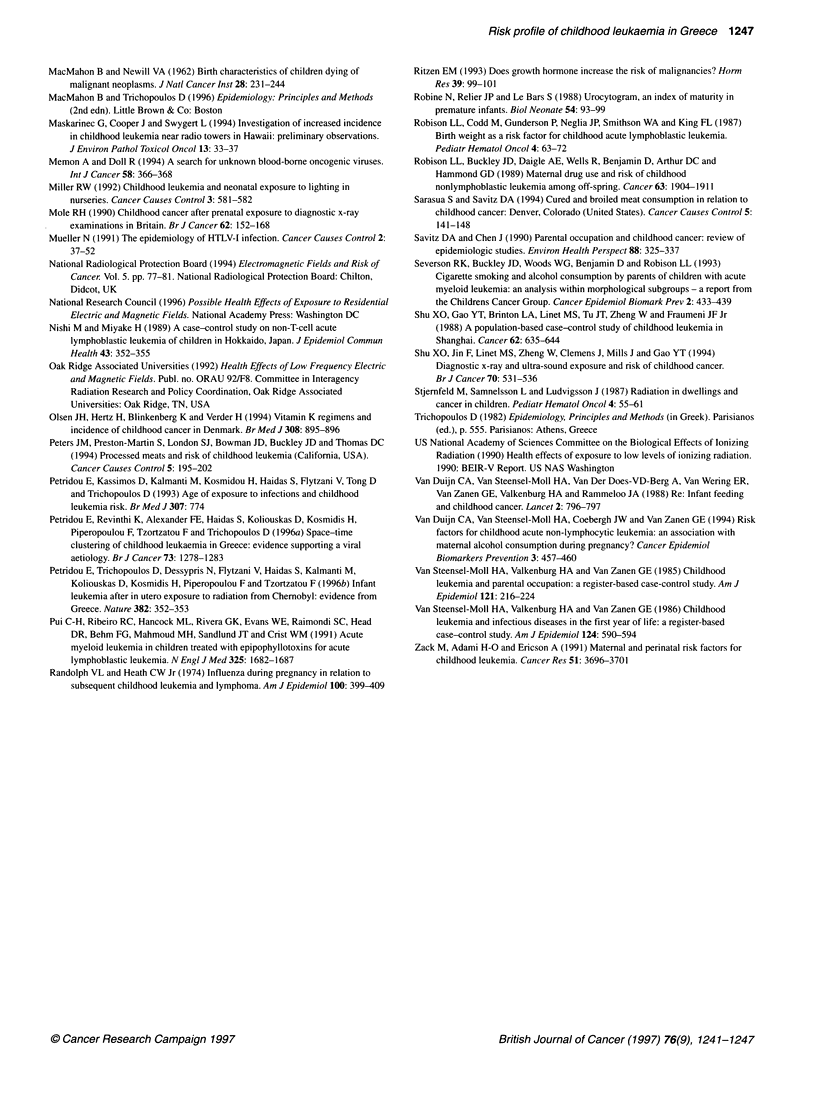


## References

[OCR_01053] Ben-Sasson S. A., Davis D. L. (1992). Neonatal exposure to protoporphyrin-activating lighting as a contributing cause of childhood acute lymphocytic leukemia.. Cancer Causes Control.

[OCR_01061] Broomhall J., May R., Lilleyman J. S., Milner R. D. (1983). Height and lymphoblastic leukaemia.. Arch Dis Child.

[OCR_01067] Bross I. D., Gibson R. (1970). Cats and childhood leukemia.. J Med.

[OCR_01069] Buckley J. D., Buckley C. M., Ruccione K., Sather H. N., Waskerwitz M. J., Woods W. G., Robison L. L. (1994). Epidemiological characteristics of childhood acute lymphocytic leukemia. Analysis by immunophenotype. The Childrens Cancer Group.. Leukemia.

[OCR_01076] Bueno de Mesquita H. B., Maisonneuve P., Moerman C. J., Walker A. M. (1992). Aspects of medical history and exocrine carcinoma of the pancreas: a population-based case-control study in The Netherlands.. Int J Cancer.

[OCR_01088] Cocco P., Rapallo M., Targhetta R., Biddau P. F., Fadda D. (1996). Analysis of risk factors in a cluster of childhood acute lymphoblastic leukemia.. Arch Environ Health.

[OCR_01093] Curnen M. G., Varma A. A., Christine B. W., Turgeon L. R. (1974). Childhood leukemia and maternal infectious diseases during pregnancy.. J Natl Cancer Inst.

[OCR_01098] Cutler J. J., Parker G. S., Rosen S., Prenney B., Healey R., Caldwell G. G. (1986). Childhood leukemia in Woburn, Massachusetts.. Public Health Rep.

[OCR_01103] Daling J. R., Starzyk P., Olshan A. F., Weiss N. S. (1984). Birth weight and the incidence of childhood cancer.. J Natl Cancer Inst.

[OCR_01107] Fasal E., Jackson E. W., Klauber M. R. (1971). Birth characteristics and leukemia in childhood.. J Natl Cancer Inst.

[OCR_01111] Fine P. E., Adelstein A. M., Snowman J., Clarkson J. A., Evans S. M. (1985). Long term effects of exposure to viral infections in utero.. Br Med J (Clin Res Ed).

[OCR_01115] Godfrey K. M., Redman C. W., Barker D. J., Osmond C. (1991). The effect of maternal anaemia and iron deficiency on the ratio of fetal weight to placental weight.. Br J Obstet Gynaecol.

[OCR_01120] Greaves M. F. (1997). Aetiology of acute leukaemia.. Lancet.

[OCR_01122] Greaves M. F., Alexander F. E. (1993). An infectious etiology for common acute lymphoblastic leukemia in childhood?. Leukemia.

[OCR_01126] Greenberg R. S., Shuster J. L. (1985). Epidemiology of cancer in children.. Epidemiol Rev.

[OCR_01134] Hartley A. L., Birch J. M., McKinney P. A., Blair V., Teare M. D., Carrette J., Mann J. R., Stiller C. A., Draper G. J., Johnston H. E. (1988). The Inter-Regional Epidemiological Study of Childhood Cancer (IRESCC): past medical history in children with cancer.. J Epidemiol Community Health.

[OCR_01130] Härö A. S. (1986). The effect of BCG-vaccination and tuberculosis on the risk of leukaemia.. Dev Biol Stand.

[OCR_01149] Jain M., Howe G. R., St Louis P., Miller A. B. (1991). Coffee and alcohol as determinants of risk of pancreas cancer: a case-control study from Toronto.. Int J Cancer.

[OCR_01154] John E. M., Savitz D. A., Sandler D. P. (1991). Prenatal exposure to parents' smoking and childhood cancer.. Am J Epidemiol.

[OCR_01158] Kaye S. A., Robison L. L., Smithson W. A., Gunderson P., King F. L., Neglia J. P. (1991). Maternal reproductive history and birth characteristics in childhood acute lymphoblastic leukemia.. Cancer.

[OCR_01172] Kinlen L. J., Clarke K., Hudson C. (1990). Evidence from population mixing in British New Towns 1946-85 of an infective basis for childhood leukaemia.. Lancet.

[OCR_01177] Kinlen L. J., Dickson M., Stiller C. A. (1995). Childhood leukaemia and non-Hodgkin's lymphoma near large rural construction sites, with a comparison with Sellafield nuclear site.. BMJ.

[OCR_01163] Kinlen L. J. (1995). Epidemiological evidence for an infective basis in childhood leukaemia.. Br J Cancer.

[OCR_01167] Kinlen L. J., Petridou E. (1995). Childhood leukemia and rural population movements: Greece, Italy, and other countries.. Cancer Causes Control.

[OCR_01182] Kneale G. W., Stewart A. M., Wilson L. M. (1986). Immunizations against infectious diseases and childhood cancers.. Cancer Immunol Immunother.

[OCR_01187] Lauritzen C., Lehmann W. D. (1966). Die Bedeutung der Steroidhormone für die Entstehung von Hyperbilirubinämie und Icterus neonatorum.. Z Kinderheilkd.

[OCR_01197] Lowengart R. A., Peters J. M., Cicioni C., Buckley J., Bernstein L., Preston-Martin S., Rappaport E. (1987). Childhood leukemia and parents' occupational and home exposures.. J Natl Cancer Inst.

[OCR_01210] MACMAHON B., NEWILL V. A. (1962). Birth characteristics of children dying of malignant neoplasms.. J Natl Cancer Inst.

[OCR_01202] MacMahon B. (1992). Is acute lymphoblastic leukemia in children virus-related?. Am J Epidemiol.

[OCR_01218] Maskarinec G., Cooper J., Swygert L. (1994). Investigation of increased incidence in childhood leukemia near radio towers in Hawaii: preliminary observations.. J Environ Pathol Toxicol Oncol.

[OCR_01223] Memon A., Doll R. (1994). A search for unknown blood-borne oncogenic viruses.. Int J Cancer.

[OCR_01227] Miller R. W. (1992). Childhood leukemia and neonatal exposure to lighting in nurseries.. Cancer Causes Control.

[OCR_01231] Mole R. H. (1990). Childhood cancer after prenatal exposure to diagnostic X-ray examinations in Britain.. Br J Cancer.

[OCR_01235] Mueller N. (1991). The epidemiology of HTLV-I infection.. Cancer Causes Control.

[OCR_01247] Nishi M., Miyake H. (1989). A case-control study of non-T cell acute lymphoblastic leukaemia of children in Hokkaido, Japan.. J Epidemiol Community Health.

[OCR_01258] Olsen J. H., Hertz H., Blinkenberg K., Verder H. (1994). Vitamin K regimens and incidence of childhood cancer in Denmark.. BMJ.

[OCR_01262] Peters J. M., Preston-Martin S., London S. J., Bowman J. D., Buckley J. D., Thomas D. C. (1994). Processed meats and risk of childhood leukemia (California, USA).. Cancer Causes Control.

[OCR_01267] Petridou E., Kassimos D., Kalmanti M., Kosmidis H., Haidas S., Flytzani V., Tong D., Trichopoulos D. (1993). Age of exposure to infections and risk of childhood leukaemia.. BMJ.

[OCR_01279] Petridou E., Trichopoulos D., Dessypris N., Flytzani V., Haidas S., Kalmanti M., Koliouskas D., Kosmidis H., Piperopoulou F., Tzortzatou F. (1996). Infant leukaemia after in utero exposure to radiation from Chernobyl.. Nature.

[OCR_01285] Pui C. H., Ribeiro R. C., Hancock M. L., Rivera G. K., Evans W. E., Raimondi S. C., Head D. R., Behm F. G., Mahmoud M. H., Sandlund J. T. (1991). Acute myeloid leukemia in children treated with epipodophyllotoxins for acute lymphoblastic leukemia.. N Engl J Med.

[OCR_01291] Randolph V. L., Heath C. W. (1974). Influenza during pregnancy in relation to subsequent childhood leukemia and lymphoma.. Am J Epidemiol.

[OCR_01295] Ritzén E. M. (1993). Does growth hormone increase the risk of malignancies?. Horm Res.

[OCR_01299] Robine N., Relier J. P., Le Bars S. (1988). Urocytogram, an index of maturity in premature infants.. Biol Neonate.

[OCR_01308] Robison L. L., Buckley J. D., Daigle A. E., Wells R., Benjamin D., Arthur D. C., Hammond G. D. (1989). Maternal drug use and risk of childhood nonlymphoblastic leukemia among offspring. An epidemiologic investigation implicating marijuana (a report from the Childrens Cancer Study Group).. Cancer.

[OCR_01303] Robison L. L., Codd M., Gunderson P., Neglia J. P., Smithson W. A., King F. L. (1987). Birth weight as a risk factor for childhood acute lymphoblastic leukemia.. Pediatr Hematol Oncol.

[OCR_01314] Sarasua S., Savitz D. A. (1994). Cured and broiled meat consumption in relation to childhood cancer: Denver, Colorado (United States). Cancer Causes Control.

[OCR_01319] Savitz D. A., Chen J. H. (1990). Parental occupation and childhood cancer: review of epidemiologic studies.. Environ Health Perspect.

[OCR_01323] Severson R. K., Buckley J. D., Woods W. G., Benjamin D., Robison L. L. (1993). Cigarette smoking and alcohol consumption by parents of children with acute myeloid leukemia: an analysis within morphological subgroups--a report from the Childrens Cancer Group.. Cancer Epidemiol Biomarkers Prev.

[OCR_01329] Shu X. O., Gao Y. T., Brinton L. A., Linet M. S., Tu J. T., Zheng W., Fraumeni J. F. (1988). A population-based case-control study of childhood leukemia in Shanghai.. Cancer.

[OCR_01334] Shu X. O., Jin F., Linet M. S., Zheng W., Clemens J., Mills J., Gao Y. T. (1994). Diagnostic X-ray and ultrasound exposure and risk of childhood cancer.. Br J Cancer.

[OCR_01339] Stjernfeldt M., Samuelsson L., Ludvigsson J. (1987). Radiation in dwellings and cancer in children.. Pediatr Hematol Oncol.

[OCR_01373] Zack M., Adami H. O., Ericson A. (1991). Maternal and perinatal risk factors for childhood leukemia.. Cancer Res.

[OCR_01357] van Duijn C. M., van Steensel-Moll H. A., Coebergh J. W., van Zanen G. E. (1994). Risk factors for childhood acute non-lymphocytic leukemia: an association with maternal alcohol consumption during pregnancy?. Cancer Epidemiol Biomarkers Prev.

[OCR_01354] van Duijn C. M., van Steensel-Moll H. A., van der Does-vd Berg A., van Wering E. R., van Zanen G. E., Valkenburg H. A., Rammeloo J. A. (1988). Infant feeding and childhood cancer.. Lancet.

[OCR_01368] van Steensel-Moll H. A., Valkenburg H. A., van Zanen G. E. (1986). Childhood leukemia and infectious diseases in the first year of life: a register-based case-control study.. Am J Epidemiol.

[OCR_01363] van Steensel-Moll H. A., Valkenburg H. A., van Zanen G. E. (1985). Childhood leukemia and parental occupation. A register-based case-control study.. Am J Epidemiol.

